# Long-term epigenetic effects of exposure to low doses of ^56^Fe in the mouse lung

**DOI:** 10.1093/jrr/rru010

**Published:** 2014-02-28

**Authors:** Etienne Nzabarushimana, Isabelle R. Miousse, Lijian Shao, Jianhui Chang, Antiño R. Allen, Jennifer Turner, Blair Stewart, Jacob Raber, Igor Koturbash

**Affiliations:** 1 Department of Environmental and Occupational Health, College of Public Health, University of Arkansas for Medical Sciences, 4301 West Markham Street, #820-11, Little Rock, 72205-7199, AR, USA; 2 Division of Radiation Health, Department of Pharmaceutical Sciences, University of Arkansas for Medical Sciences, 4301 West Markham Street, #820-11, Little Rock, 72205-7199, AR, USA; 3 Department of Behavioral Neuroscience, ONPRC, Oregon Health and Science University, 3181 S.W. Sam Jackson Park Rd, Portland, 97239-3098, OR, USA; 4 Department of Neurology, ONPRC, Oregon Health and Science University, 3181 S.W. Sam Jackson Park Rd, Portland, 97239-3098, OR, USA; 5 Division of Cancer Biology and Radiobiology, and Division of Neuroscience, ONPRC, Oregon Health and Science University, 3181 S.W. Sam Jackson Park Rd, Portland, 97239-3098, OR, USA

**Keywords:** heavy iron ions, epigenetics, DNA methylation, repetitive elements, pulmonary fibrosis, lung cancer

## Abstract

Despite significant progress, the long-term health effects of exposure to high charge (Z) and energy (E) nuclei (HZEs) and the underlying mechanisms remain poorly understood. Mouse studies show that space missions can result in pulmonary pathological states. The goal of this study was to evaluate the pro-fibrotic and pro-carcinogenic effects of exposure to low doses of heavy iron ions (^56^Fe) in the mouse lung. Exposure to ^56^Fe (600 MeV; 0.1, 0.2 and 0.4 Gy) resulted in minor pro-fibrotic changes, detected at the beginning of the fibrotic phase (22 weeks post exposure), which were exhibited as increased expression of chemokine *Ccl3,* and interleukin *Il4*. Epigenetic alterations were exhibited as global DNA hypermethylation, observed after exposure to 0.4 Gy. *Cadm1*, *Cdh13*, *Cdkn1c*, *Mthfr* and *Sfrp1* were significantly hypermethylated after exposure to 0.1 Gy, while exposure to higher doses resulted in hypermethylation of *Cdkn1c* only. However, expression of these genes was not affected by any dose. Congruently with the observed patterns of global DNA methylation, DNA repetitive elements were hypermethylated after exposure to 0.4 Gy, with minor changes observed after exposure to lower doses. Importantly, hypermethylation of repetitive elements coincided with their transcriptional repression. The findings of this study will aid in understanding molecular determinants of pathological states associated with exposure to ^56^Fe, as well as serve as robust biomarkers for the delayed effects of irradiation. Further studies are clearly needed to investigate the persistence and outcomes of molecular alterations long term after exposure.

## INTRODUCTION

Understanding extended health effects of exposure to high charge (Z) and energy (E) nuclei (HZEs) is important for long-term space missions. Pulmonary fibrosis is a well-documented consequence of radiation exposure [1], and space radiation may also alter pulmonary function. Indeed, recent experimental evidence indicated the presence of molecular alterations associated with pulmonary fibrosis in mice shortly after a space mission [2].

About one-third of all the cancers attributable to radiation in atomic bomb survivors and nuclear reactor workers are lung cancers [3, 4]. Also, epidemiological studies demonstrate that lung cancer is the most frequent cancer associated with radiotherapy and accounts for almost a quarter of all radiotherapy-induced secondary malignancies [5]. Moreover, lung cancer is the largest potential cancer risk for astronauts [6].

Studies, performed on immortalized human bronchial cells (BEP2D) have shown that cells become tumorigenic after exposure to 1 GeV/nucleon of heavy iron ions (^56^Fe), displaying mutations in the *p53* gene but lacking other genetic alterations, such as *ras* mutations and deletion in the *p16*^*INK4A*^ gene [7]. This suggests involvement of other, probably epigenetic, alterations in HZE-induced lung carcinogenesis.

DNA methylation is an important epigenetic mechanism and is critical for proper expression of genetic information and silencing of DNA repetitive elements. Alterations in DNA methylation may lead to aberrant expression of oncogenes and repetitive elements, and silencing of tumor-suppressor genes, resulting in genomic instability and cancer [8]. The role of epigenetic alterations in lung carcinogenesis has become increasingly recognized [9]. Studies have shown that radiation-induced lung adenocarcinomas are associated with an increased frequency of genes inactivated via promoter hypermethylation [10]. Additionally, *p16^INK4A^* hypermethylation was detected in the lung tumors of workers exposed to plutonium [11]. Early appearance of epigenetic alterations during carcinogenesis, together with their persistence, makes them valuable biomarkers of carcinogenic exposure [12].

Despite significant progress in the field, the long-term health effects of exposure to high-LET radiation, and the underlying molecular alterations, remain poorly understood. Therefore, the goal of this study was to evaluate the molecular endpoints associated with pulmonary fibrosis and lung carcinogenesis long-term (22 weeks) after exposure to low doses of ^56^Fe irradiation.

## MATERIALS AND METHODS

### Animals and radiation exposures

Six-month-old male C57BL/6J mice (*n* = 40) purchased from the Jackson Laboratory (Bar Harbor, ME) were shipped to Brookhaven National Laboratories (BNL) in Upton, NY. After a one-week acclimation period, the mice were either sham irradiated or received whole-body irradiation (^56^Fe 600 MeV/n; 0.1, 0.2 or 0.4 Gy, *n* = 10 mice per group). One week after irradiation, the mice were shipped to Oregon Health and Science University (OHSU). At BNL and OHSU, the mice were housed under a constant 12 h light:dark cycle. Food (PicoLab Rodent Diet 20, No. 5053; PMI Nutrition International, St Louis, MO) and water were provided *ad libitum*. Animals were killed by cervical dislocation 22 weeks after irradiation; lungs were excised and immediately frozen in liquid nitrogen. All procedures were approved by the Institutional Animal Care and Use Committees at OHSU and BNL.

### Quantitative reverse transcription polymerase chain reaction

Total RNA was extracted from lungs (20 mg per sample) using the AllPrep DNA/RNA extraction kit (QIAGEN, Valencia, CA) according to the manufacturer's protocol. Levels of gene transcripts were determined by quantitative reverse transcription polymerase chain reaction (qRT-PCR) using TaqMan Gene Expression Assays (Life Technologies, Grand Island, NY, and Integrated DNA Technologies, Coralville, IA) according to the manufacturer's protocol. The mRNA abundance of repetitive elements was determined by qRT-PCR, as previously described [13]. Briefly, cDNA was synthesized from 1 µg total RNA. The relative level of mRNA for each repetitive element was determined using the 2^ΔΔCt^ method. The results were normalized to *Gapdh* and *β-actin* values and represented as fold change relative to those from control mice.

### Quantification analysis of 5-methylcytosine

Total DNA was extracted as described above. Whole genome CpG methylation was assessed using a commercially available fluorescence-based immunoassay according to the manufacturer's protocol (Epigentek, Farmingdale, NY).

### Analysis of gene-specific methylation

Gene-specific methylation was measured by Mouse Lung Cancer DNA Methylation PCR Array (SABiosciences, Frederick, MD) according to the manufacturer's protocol.

### Analysis of the methylation status of DNA repetitive elements

The methylation status of repetitive elements was determined by methylation-sensitive McrBC-qPCR assay as previously described [13].

### Statistical analysis

All statistical analyses were performed using SAS 9.3 (SAS Institute Inc., Cary, NC) and GraphPad Prism 6 (GraphPad Software, San Diego, CA). The data are presented as mean ± standard error of 10 independent samples per dose. The dose-effect was statistically assessed using ROUT's test (to eliminate outliers) and one-way ANOVA and Kruskal–Wallis methods, followed by Tukey's test and the Jonckheere trend test when appropriate.

## RESULTS

### Analysis of expression of genes associated with development of pulmonary fibrosis

**Fig. 1.**
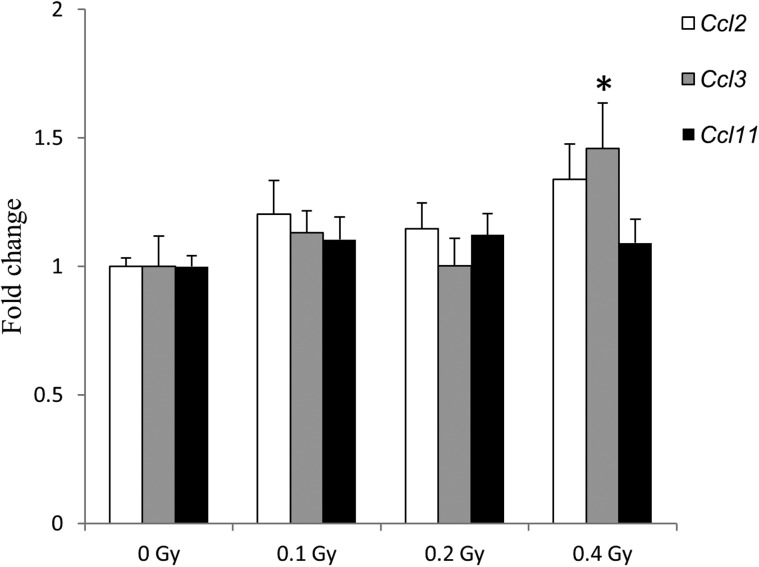
Expression of fibrosis-associated genes 22 weeks after exposure to ^56^Fe. The differential gene expression was determined by quantitative RT-PCR. Data are presented as mean ± SEM (*n* = 10). Asterisks (*) denote significant (*P* < 0.05) difference from control.

A short-term space mission resulted in pro-fibrotic changes in mouse lungs detected shortly after landing [2]. Therefore, we sought to determine whether a single exposure to ^56^Fe may result in long-lasting fibrotic changes in the mouse lung. For this purpose, we measured the expression of a panel of genes previously reported to be aberrantly expressed at the beginning of the fibrotic phase (Supplementary Table 1) [2, 14]. As shown in Fig. 1, an increase in *Ccl3* expression was observed after exposure to 0.4 Gy of ^56^Fe (1.5-fold, *P* < 0.05), but no alterations were seen at lower doses. Exposure to ^56^Fe resulted in an insignificant increase in *Il4* expression after exposure to 0.2 and 0.4 Gy, (1.9-fold, *P*-value 0.1; and 1.4-fold, *P*-value 0.1, respectively) (Supplementary Table 1).

### Effects of exposure to ^56^Fe on global DNA methylation and DNA methylation machinery

**Fig. 2.**
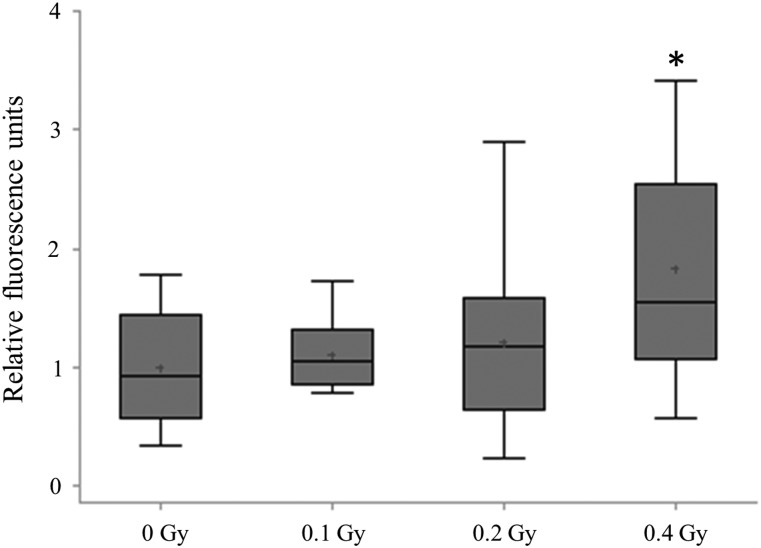
Analysis of global DNA methylation. Levels of 5-methylcytosine in the mouse lung. Data are presented as mean ± SEM (*n* = 10). Asterisks (*) denote significant (*P* < 0.05) difference from control.

Alterations in DNA methylation have been reported in lung cancer, at early stages of lung carcinogenesis, and after exposure to lung carcinogens [9, 15]. Therefore, we next addressed global DNA methylation by analyzing the levels of 5-methylcytosine (5-mC) in the exposed and sham-irradiated animals. We identified a dose-dependent increase in the quantity of 5-mC in the mouse lung that reached significance (1.6-fold, *P* < 0.05) after exposure to 0.4 Gy (Fig. 2). Concurrently, radiation exposure did not lead to aberrant expression of the DNA methylation machinery—*Dnmt1*, *Dnmt3a* and *Dnmt3b* (Supplementary Fig. 1).

### Analysis of gene-specific methylation

To validate the origin of the hypermethylation, we first evaluated promoter methylation of 22 genes that were previously reported to be hypermethylated with a high frequency in lung cancer. The most pronounced changes were observed after exposure to 0.1 Gy; five genes (*Cadm1*, *Cdh13*, *Cdkn1c*, *Mthfr* and *Sfrp1*) were significantly (*P*-value < 0.05) hypermethylated (Table 1). Only one gene (*Cdkn1c*) was significantly hypermethylated after exposure to 0.2 or 0.4 Gy, suggesting that the lowest dose exposure may have more profound effects on gene-specific methylation.

**Table 1.** Gene-specific methylation 22 weeks after exposure to ^56^Fe

Gene name	0 Gy		0.1 Gy		0.2 Gy		0.4 Gy	
	% mCpG	% uCpG	% mCpG	% uCpG	% mCpG	% uCpG	% mCpG	% uCpG
*Cadm1*	0.1	99.9	20.2	79.8	0.1	99.9	0	100
*Sfrp1*	0	100	17.7	82.3	0	100	0.1	99.9
*Cdkn1c*	7.8	92.2	38.5	61.5	11.5	88.5	13.2	86.8
*Cdh13*	2.7	97.3	17.9	82.1	1.9	98.1	2.5	97.5
*Mthfr*	0.1	99.9	32.6	67.4	0.1	99.9	0.1	99.9

% mCpG = percentage of methylated CpG sites, % uCpG = percentage of unmethylated CpG sites.

Hypermethylation of promoter regions of tumor-suppressor genes is often associated with their transcriptional silencing [8, 16]. To identify whether hypermethylation observed 22 weeks after exposure to ^56^Fe is associated with transcriptional alterations, we measured the expression of *Cadm1*, *Cdkn1c* and *Mthfr*. Additionally, we measured the expression of three selected genes whose methylation was not affected by the exposure (*Apc*, *Cdkn2a* and *Rassf1*). No significant differences in expression of any of these genes were identified (Supplementary Table 1).

### Effects of ^56^Fe exposure on repetitive elements

**Fig. 3.**
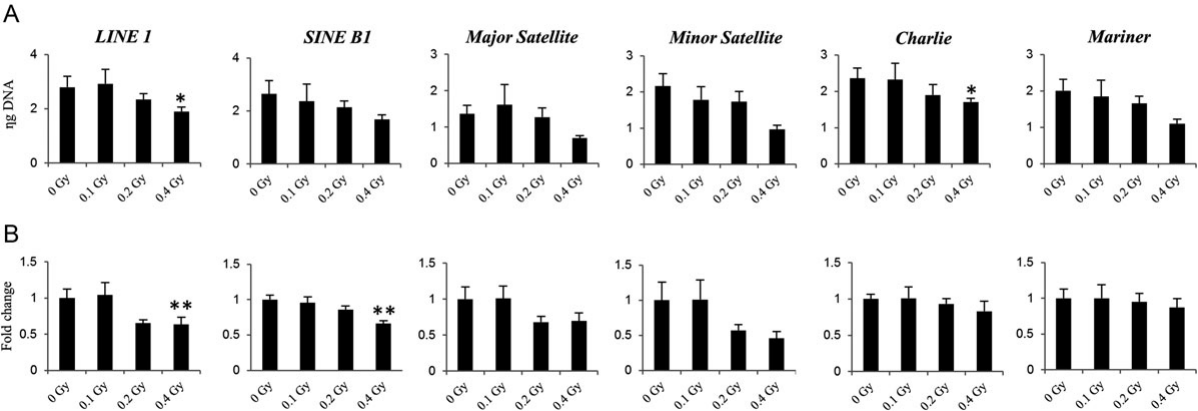
Effects of ^56^Fe exposure on repetitive elements. (**A**) Methylation of DNA repetitive elements as measured by methylation-sensitive McrBC-qPCR assay. Data are presented as mean ± SEM (*n* = 10). The means are inversely correlated to the extent of individual repetitive elements methylation. Asterisks (*) denote significant (*P* < 0.05) difference from control (Tukey's test). (**B**) The differential expression of repetitive elements was determined by quantitative RT-PCR. Data are presented as mean ± SEM (*n* = 10). Asterisks (*) denote significant (*P* < 0.05) and (**) (*P* < 0.01) difference from control (Tukey's test).

Next, we addressed the methylation of DNA repetitive elements that constitute nearly half of the mammalian genomes and often reflect the status of global genomic methylation. Exposure to 0.4 Gy of ^56^Fe resulted in significant hypermethylation in LINE1 (*P-*value 0.03) and Charlie (*P-*value 0.05), near significant hypermethylation in minor satellites and Mariner (*P-*value 0.06), and trends towards hypermethylation in SINE B1 and major satellites (*P-*value 0.09) (Fig. 3A).

Accumulating evidence suggests that DNA methylation serves as a repressive mechanism to silence the expression of repetitive elements. To identify whether the observed repetitive elements radiation-associated *de novo* hypermethylation may negatively affect transcription, we measured their expression using qRT-PCR. The expression of LINE1 and SINE B1, the two most abundant repetitive elements in mammalian genomes, was significantly diminished after exposure to 0.4 Gy (1.8-fold, *P-*value 0.007; and 1.7-fold, *P-*value 0.0024, respectively). Insignificant loss of expression was also observed for major and minor satellites and Charlie (Fig. 3B).

## DISCUSSION

In the current study, we evaluated the molecular effects associated with two major pathological states in the lung—pulmonary fibrosis and cancer—after exposure to ^56^Fe. Subtle alterations in the expression of specific genes, suggesting minor pro-fibrotic changes in the lungs of animals, were observed after a 0.4-Gy dose, with lower doses failing to result in any notable effect. This finding suggests that the utilized doses are probably too low to cause significant fibrotic changes in the lung. It is also possible that other factors, such as combined exposure to different types of radiation (^56^Fe, other heavy ions and protons) and weightlessness may contribute to the pulmonary fibrosis.

We identified a significant (1.6-fold, *P* < 0.05) hypermethylation effect in mice exposed to 0.4 Gy of ^56^Fe. Consistent with our findings, others have noted hypermethylation in various cell lines (RKO, AG01522 and GM10115) at 16–20 population doublings post ^56^Fe and protons exposure [15, 17]. In contrast, previous studies involving low-LET radiation reported radiation-induced [18] and carcinogenesis-associated global DNA hypomethylation [8]. These findings suggest that high- and low-LET radiation might have differential effects on DNA methylation. Indeed, in our study, the key players in the regulation of DNA methylation that are frequently deregulated after exposure to low-LET radiation remained unchanged after exposure to high-LET radiation.

Tumor-suppressor gene-specific hypermethylation is a common feature of cancers, including radiation-induced lung adenocarcinomas [10]. It has been proposed that changes in individual gene DNA methylation are the driving events in tumorigenesis and can precede genetic mutations [19]. In our study, only a small fraction of genes (5 out of 22) were found hypermethylated after exposure to 0.1 Gy of ^56^Fe, and this was not associated with changes in their expression. Our results are in good agreement with a recent study that reported exposure to 0.1 Gy of ^56^Fe resulted in cyclic alterations in gene-specific DNA methylation in the mouse lung that did not correlate with changes in the associated mRNA levels [20]. As in our study, a lack of gene-specific methylation changes was observed after exposure to higher doses [20]. These findings, however, are worthy of longitudinal investigation, since DNA hypermethylation is an early event in carcinogenesis, and it appears that the number and extent of aberrant hypermethylation events are progressively increased from a precancerous to a cancerous condition [8, 15, 21].

High-LET radiation also induced hypermethylation of DNA repetitive elements. Our previous low-LET studies have clearly demonstrated the loss of DNA methylation in repetitive elements following exposure to radiation [22]. DNA methylation is thought to be important in silencing repetitive elements, preventing reactivation of gene expression and potential development of genomic instability. On the other hand, the role of repetitive elements has been recently revisited, suggesting their role in the regulation of alternative mRNA processing and in generating small regulatory RNAs [23]. It has been proposed that alterations in gene expression could be caused via alterations in the chromatin structure in inactive LINE1 that allows for transcriptional interference by neighboring enhancers and silencers [24]. Also, a recent study has indicated the association between the hypermethylation of repetitive elements and the early-onset of colorectal cancer [25]. These findings suggest that hypermethylation of repetitive elements might be critical in tumor development.

This is the first study, to our knowledge, that evaluates the long-term molecular effects of exposure to ^56^Fe in lung tissue. Importantly, the doses used in our study are in a similar range to that which could be received during space missions. The findings of this study will aid in understanding molecular determinants of pathological states associated with exposure to ^56^Fe, as well as potentially identifying biomarkers for the delayed effects of irradiation. Further studies designed to investigate the molecular alterations and methylation status of repetitive elements and their relationship to transcriptional silencing, especially using different mouse strains, are clearly needed to extend these initial findings.

## SUPPLEMENTARY DATA

Supplementary data is available at the *Journal of Radiation Research* online.

## FUNDING

The work was supported in part by the National Aeronautics and Space Administration (NNJ12ZSA001N to J.R.), the NIH/UAMS Clinical and Translational Science Award (UL1TR000039 and KL2TR000063), and the Arkansas Biosciences Institute (I.K.).

## Supplementary Material

Supplementary Data
