# Supplementary material for: Long-term epigenetic effects of exposure to low doses of 56Fe in the mouse lung
Source: J Radiat Res. 2014 Feb 28;55(4):823–8. doi: 10.1093/jrr/rru010 (PMC4100002; doi:10.1093/jrr/rru010)
Supplement: Supplementary Data [file supp_55_4_823__index.html]

Long-term epigenetic effects of exposure to low doses of 56Fe in the mouse lung — Supplementary Data 

# Long-term epigenetic effects of exposure to low doses of 56Fe in the mouse lung

## Supplementary Data

Supplementary Data

**Files in this Data Supplement:**

- Supplementary Data - Docx file
