# Supplementary material for: Long-term epigenetic effects of exposure to low doses of 56Fe in the mouse lung
Source: J Radiat Res. 2014 Feb 28;55(4):823–8. doi: 10.1093/jrr/rru010 (PMC4100002; doi:10.1093/jrr/rru010)

Supplementary Table 1. Gene-expression analysis 22 weeks after exposure to ^56^Fe. The differential gene expression was determined by quantitative RT-PCR. Data are presented as mean fold change from control ± SD (n=10). ). Asterisks (*) denote significant (*p*<0.05) difference from control.

| **Gene name** | **0.1 Gy** | **0.2 Gy** | **0.4 Gy** |
| --- | --- | --- | --- |
| *Acta2* | 1.0 | 1.2 | 1.1 |
| *Ccl2* | 1.2 | 1.2 | 1.3 |
| *Ccl3* | 1.1 | 1 | 1.5* |
| *Ccl11* | 1.1 | 1.1 | 1.1 |
| *Col1a1* | 1 | 1 | 1.1 |
| *Col3a1* | 1 | 1 | 1.2 |
| *Ctgf* | 1.3 | 1.2 | 1.2 |
| *Tgfβ1* | 1 | 1 | 1 |
| *Mmp2* | 0.9 | 1 | 1 |
| *Mmp7* | 0.7 | 0.8 | 0.9 |
| *Mmp9* | 0.7 | 0.8 | 0.8 |
| *Il3* | 1.3 | 2.5 | 1.9 |
| *Il4* | 1.1 | 1.9 | 1.4 |
| *Cadm1* | 0.9 | 1.1 | 1.1 |
| *Cdkn1c* | 1.1 | 0.9 | 0.9 |
| *Mthfr* | 0.9 | 1.1 | 0.8 |
| *Apc* | 1 | 1 | 0.9 |
| *Cdkn2a* | 1 | 0.9 | 1.1 |
| *Rassf1* | 1 | 0.9 | 1 |

Supplementary Figure 1. Effects of ^56^Fe exposure on DNA methylation machinery. The differential expression of *Dnmt1*, *Dnmt3a*, and *Dnmt3b* was determined by quantitative RT-PCR. Data are presented as mean ± SD (n=10).


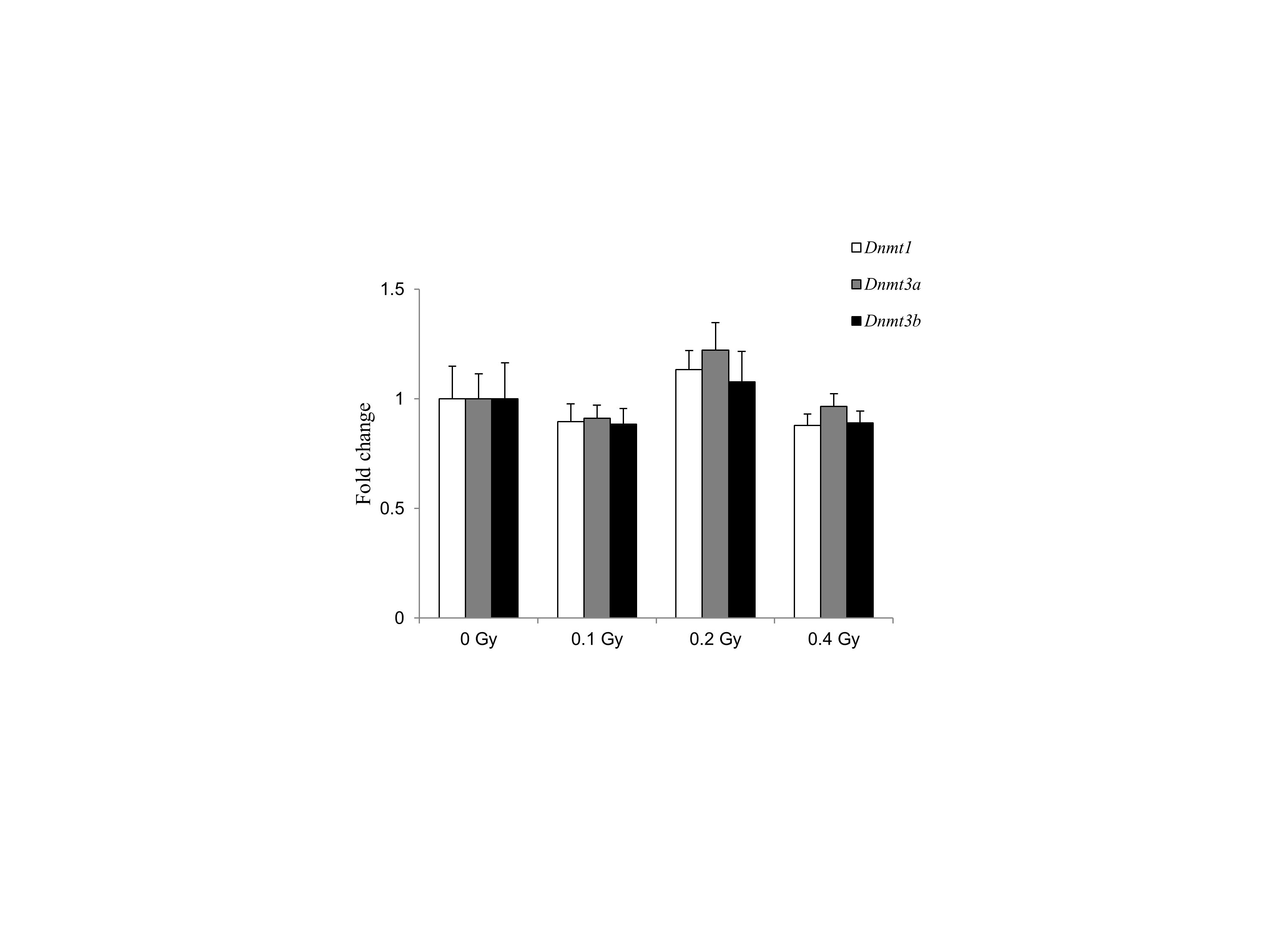

Supplement: Supplementary Data [file supp_rru010_rru010supp.docx]
